# Lack of Charge Interaction in the Ion Binding Site Determines Anion Selectivity in the Sodium Bicarbonate Cotransporter NBCe1

**DOI:** 10.3390/ijms23010532

**Published:** 2022-01-04

**Authors:** Soojung Lee, Jason Lin, Inyeong Choi

**Affiliations:** Department of Cell Biology, Emory University School of Medicine, Atlanta, GA 30322, USA; Soojung.lee@gatech.edu (S.L.); Jason.lin@emory.edu (J.L.)

**Keywords:** sodium bicarbonate transporter, NBCe1, SLC4A, ion transport, anion selectivity, structure function

## Abstract

The Na/HCO_3_ cotransporter NBCe1 is a member of SLC4A transporters that move HCO_3_^−^ across cell membranes and regulate intracellular pH or transepithelial HCO_3_ transport. NBCe1 is highly selective to HCO_3_^−^ and does not transport other anions; the molecular mechanism of anion selectivity is presently unclear. We previously reported that replacing Asp^555^ with a Glu (D555E) in NBCe1 induces increased selectivity to other anions, including Cl^−^. This finding is unexpected because all SLC4A transporters contain either Asp or Glu at the corresponding position and maintain a high selectivity to HCO_3_^−^. In this study, we tested whether the Cl^−^ transport in D555E is mediated by an interaction between residues in the ion binding site. Human NBCe1 and mutant transporters were expressed in *Xenopus* oocytes, and their ability to transport Cl^−^ was assessed by two-electrode voltage clamp. The results show that the Cl^−^ transport is induced by a charge interaction between Glu^555^ and Lys^558^. The bond length between the two residues is within the distance for a salt bridge, and the ionic strength experiments confirm an interaction. This finding indicates that the HCO_3_^−^ selectivity in NBCe1 is established by avoiding a specific charge interaction in the ion binding site, rather than maintaining such an interaction.

## 1. Introduction

NBCe1 is a membrane protein that mediates electrogenic Na^+^-HCO_3_^−^ and/or CO_3_^2−^ transport across cell membrane and regulates intracellular and extracellular pH, as well as transepithelial HCO_3_^−^ transport in many cells [1,2,3,4]. NBCe1 was first physiologically identified in the kidney proximal tubules [5], where it is responsible for reabsorbing two thirds of filtered HCO_3_^−^. NBCe1 is highly selective to HCO_3_^−^ and does not transport other anions, including Cl^−^ [5]. The gene encoding NBCe1 was expression-cloned in the late 1990s by Romero et al. [1]; since then, it has provided valuable information on the molecular and cellular physiology of Na/HCO_3_ transport mediated by NBCe1 and its family proteins, collectively the Na^+^-coupled bicarbonate transporters, in humans. NBCe1 exists as multiple variants, due to different N- and C-terminal sequences, and each variant differs in tissue expression, intrinsic functional properties, and regulation [6]. The transporter has a Na^+^:HCO_3_^−^ stoichiometry of 1:3 in renal proximal tubules and 1:2 in other cells, as well as in heterologous expression systems. Overall, NBCe1 plays an important role in the physiology and pathophysiology of many different organs, such as kidneys, heart, brain, eyes, enamel organs, and intestines [7,8,9,10].

The cryoEM structure of NBCe1 [11] has provided details on the protein structure and amino acid residues responsible for ion transport. NBCe1 is a homodimer with each monomer, consisting of 14 transmembrane segments (TMs), extracellular loops, and cytoplasmic regions. TMs 5–7 and 12–14 form the gate domain and TMs 1–4 and 8–11 form the core domain, while the cavity between these two domains houses an ion access pathway, through which Na^+^ and CO_3_^2−^ (HCO_3_^−^) move. The ion accessibility pathway is formed by TMs 1, 3, 5, 8, 10, 12, and a short loop connecting TM13 and TM14, and the diameter along the pathway varies, ranging from >12 Å in the entrance region to ~2 Å diameter in the middle of the protein. Many amino acid residues lining the pathway were previously recognized as residues critical for transport function by mutagenesis studies [12,13,14,15,16,17]. Together with the structures of the Na^+^-driven Cl/HCO_3_^−^ exchanger NDCBE [18] and Cl/HCO_3_ changer AE1 [19], NBCe1 structure has greatly advanced our understanding of the molecular mechanisms underlying Na^+^, Cl^−^, HCO_3_^−^, and CO_3_^2−^ transport via SLC4A bicarbonate transporters.

Recently, Zhekova et al. [20] performed site identification by ligand competitive saturation (SILCS) mapping of the binding pockets in human AE1 and NBCe1, followed by molecular dynamics (MD) simulations, and proposed two putative anion binding sites in the ion accessibility pathway of the proteins: central site S1 and entrance site S2. Site S2 serves as a transient binding site, to attract anions from the surrounding solution before ion movement to site S1, where the anion binding induces a protein conformational change for ion translocation. In NBCe1, site S2 is composed of Asp^555^, Lys^558^, Lys^559^, and Lys^562^, all of which are in TM 5, and site S1 has residues from multiple TMs and loops. We have previously reported that substituting Asp^555^ with a Glu (D555E) causes the transporter to be permissive to other anions, including Cl^−^, NO_3_^−^, SCN^−^, I^−^, and Br^−^ [17]. D555E maintains favorable access to HCO_3_^−^; thus, it produces an outward current (*I*_NBC_) when HCO_3_^−^ is available but a Cl^−^ current (*I*_Cl_) when HCO_3_^−^ is unavailable. This modified selectivity should be related to a geometrical difference in the carboxyl side chain of Asp vs. Glu, due to an additional carbon backbone. On the other hand, all members of Na^+^-coupled bicarbonate transporters contain either Asp or Glu at the corresponding site, implicating that the geometrical difference in the carboxyl side chain is not the only cause for altered anion selectivity and an additional mechanism should be involved. Elucidating that mechanism will help us understand how bicarbonate transporters selectively transport HCO_3_^−^, while excluding other anions.

In this study, we investigated the effect of charge interactions between residues in site 2 on anion selectivity. Candidate residues were changed by TM5 replacement and site-directed mutagenesis, and the mutant transporters were expressed in *Xenopus* oocytes and subjected to recordings of *I_Cl_*, *I*_NBC_, and intracellular pH (pH_i_). The results show that *I*_Cl_ is induced by a charge interaction between residues in site 2. The two residues involved in the interaction are not simultaneously present in any member of the SLC4A bicarbonate transporters; thus, the HCO_3_^−^ selectivity is maintained by avoiding a charge interaction in the ion binding site, located at the entrance of the ion accessibility pathway. We also find that Na^+^ is required for HCO_3_^−^ access to the transporter, consistent with a conventional concept of a Na^+^ prerequisite for substrate movement in secondary active transporters.

## 2. Results

### 2.1. I_Cl_ Produced by D555E

**Figure 1:**
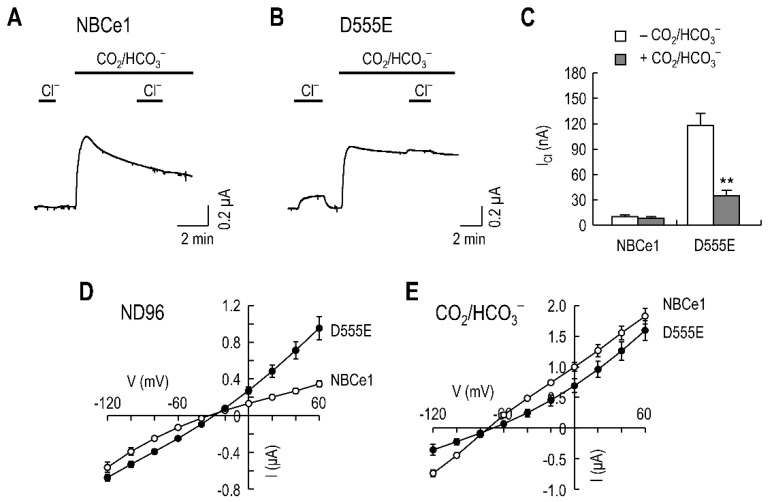
D555E produces *I*_Cl_. (**A**) Representative *I*_Cl_ and *I*_NBC_ produced by NBCe1. An oocyte expressing NBCe1 was superfused with modified Cl^−^-free ND96 until the basal current became stable, and then exposed to 71 mM Cl^−^ before and after switching solutions equilibrated with 5% CO_2_, 25 mM HCO_3_^−^. The holding potential was −60 mV. (**B**) Representative *I*_Cl_ and *I*_NBC_, produced by D555E. The recording was performed, as described in (**A**). (**C**) Mean *I*_Cl_, in the absence and presence of CO_2_/HCO_3_^−^. *I*_Cl_ was measured when the current reached steady-state after Cl^−^ application (*n* = 6/group); ** *p* < 0.01 compared to *I*_Cl_ in the absence of CO_2_/HCO_3_^−^. (**D**,**E**) *I–V* relationships of NBCe1 and D555E for *I*_Cl_ in ND96 (**D**) and CO_2_/HCO_3_^−^ (**E**). Currents were obtained by a step voltage command from −120 to +60 mV with 20 mV increments (*n* = 5/group).

To record *I*_Cl_, produced by D555E, we expressed human NBCe1 and mutant D555E in oocytes and applied them with 71 mM Cl^−^ during superfusion of Cl^−^-free solution. Figure 1A shows a representative current trace, produced by NBCe1, in voltage clamp (the holding potential of −60 mV). NBCe1 did not produce measurable *I*_Cl_, in response to bath Cl^−^, but produced an outward *I*_NBC_ upon exposure to 5% CO_2_, 25 mM HCO_3_^−^, consistent with its electrogenic Na/HCO_3_ cotransport activity. In contrast, D555E produced an outward *I*_Cl_, in response to Cl^−^ (Figure 1B). *I*_Cl_ was markedly decreased in the presence of CO_2_/HCO_3_^−^, consistent with our previous report [17] that D555E produces *I*_Cl_, which can be inhibited by HCO_3_^−^. Mean *I*_Cl_ from other oocytes (*n* = 6/group) is summarized in Figure 1C. On average, 70% of *I*_Cl_ produced by D555E was reduced in the presence of CO_2_/HCO_3_^−^ (*p* < 0.01, two-way ANOVA). In other experiments, we then determined *I–V* relationships for *I*_Cl_ and *I*_NBC_ to compare the current responses at different voltages in NBCe1 vs. D555E. As shown in Figure 1D, D555E evoked large outward currents at positive potentials in ND96 solution containing 96 mM Cl^−^ (*p* < 0.01, *n* = 5), reflecting Cl^−^ influx. However, in CO_2_/HCO_3_^−^ solution (Figure 1E), NBCe1 produced larger outward *I*_NBC_ than D555E at positive potentials (*p* < 0.05, *n* = 5). The two *I–V* curves were parallel to each other in the outward direction, as they are *I*_NBC_. The curves crossed at a negative potential (approximately −80 mV), probably due to Cl^−^ efflux via D555E in the inward direction.

### 2.2. Na^+^ Prerequisite for HCO_3_^−^ to Access Its Binding Site

**Figure 2:**
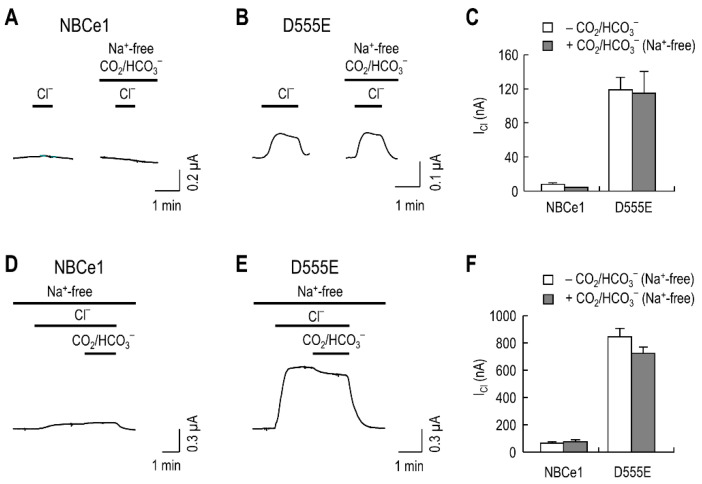
D555E-mediated *I*_Cl_ is produced in Na^+^-free CO_2_/HCO_3_^−^ solution. (**A**,**B**) Representative *I*_Cl_ produced by NBCe1 and D555E. Recording *I*_Cl_ was performed in the absence of CO_2_/HCO_3_^−^ and then repeated in Na^+^-free solution equilibrated with 5% CO_2_, 25 mM HCO_3_^−^. (**C**) Mean *I*_Cl_, produced by NBCe1 and D555E, in the absence of CO_2_/HCO_3_^−^ vs. the presence of Na^+^-free CO_2_/HCO_3_^−^ (*n* = 5 NBCe1 and 10 D555E). (**D**,**E**) Effects of Na^+^-free CO_2_/HCO_3_^−^ on *I*_Cl_, produced by NBCe1 and D555E. *I*_Cl_ was measured before and after CO_2_/HCO_3_^−^ was applied. All solutions lacked Na^+^. (**F**) Mean *I*_Cl_ before and after application of CO_2_/HCO_3_^−^ under the Na^+^-free condition (*n* = 5/group). The holding potential was −60 mV in all experiments.

The above results reveal that Cl^−^ transport by D555E is less favorable than HCO_3_^−^ transport, when both ions are present in the bath. To determine whether this feature depends on Na^+^, we performed two sets of experiments. In the first set of experiments, we recorded *I*_Cl_ in Na^+^-free CO_2_/HCO_3_^−^ solution and tested whether *I*_Cl_ could be reduced under this condition. Representative recordings of *I*_Cl_, produced by NBCe1 and D555E, are shown in Figure 2A,B. In contrast to NBCe1, D555E produced *I*_Cl_ in the absence of CO_2_/HCO_3_^−^ and, more importantly, in the Na^+^-free CO_2_/HCO_3_^−^ solution. The current amplitudes were similar in both solutions, indicating that HCO_3_^−^ has negligible effect on *I*_Cl_ under the Na^+^-free condition. Figure 2C is a comparison of the mean *I*_Cl_ between groups in these two solutions from other oocytes (*n* = 5 NBCe1 and 10 D555E). No significant difference was observed within groups. In the second set of experiments, we induced *I*_Cl_ in Na^+^-free solution and tested whether the induced *I*_Cl_ could remain after CO_2_/HCO_3_^−^ application under the continued Na^+^-free condition. As shown in Figure 2D,E, whereas NBCe1 had no *I*_Cl_, D555E produced *I*_Cl_ under the Na^+^-free condition, regardless of bath CO_2_/HCO_3_^−^. A slight decrease after CO_2_/HCO_3_^−^ application is probably due to Cl^−^ mismatch between solutions. Consistent with this result, comparison of mean *I*_Cl_ (*n* = 5/group), before and after CO_2_/HCO_3_^−^ application, resulted in no significant difference (Figure 2F). Conclusively, the results from the two sets of experiments demonstrate D555E preference to Cl^−^ over HCO_3_^−^ in the absence of Na^+^, implying that Na^+^ is required for HCO_3_^−^ to access its binding site.

### 2.3. Lack of I_Cl_ in the TM5-Replaced Chimeric Transporter

**Figure 3:**
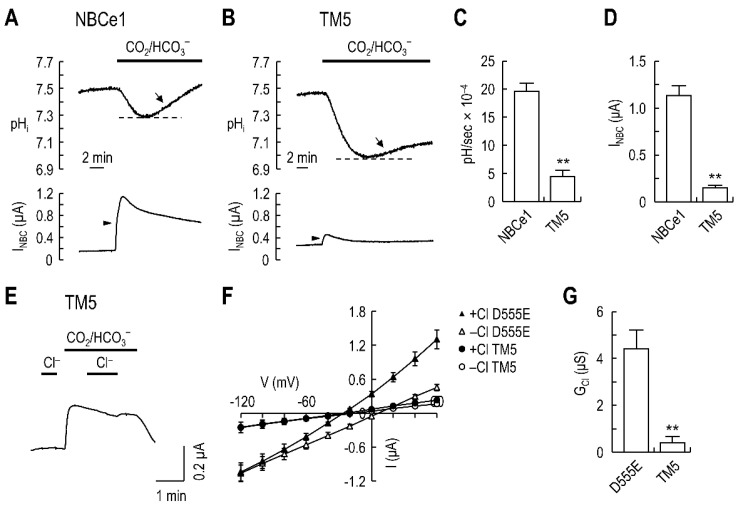
The TM5-replaced chimeric transporter does not induce *I*_Cl_. (**A**) Representative pH_i_ and *I*_NBC_ produced by NBCe1. pH_i_ and *I*_NBC_ were simultaneously recorded in voltage clamp. pH_i_ recovery (*arrow*) from a CO_2_-induced acidification and *I*_NBC_ (*arrowhead*) upon CO_2_/HCO_3_^−^ application are hallmarks for electrogenic Na/HCO_3_ transport. (**B**) Representative pH_i_ and I_NBC_, produced by the TM5 chimeric transporter. pH_i_ recovery and *I*_NBC_ characteristic for electrogenic transport are shown. (**C**) Mean pH_i_ recovery rate, dpH/dt (pH change per sec). The rate was determined by drawing a line during the first 4 min of recovery from acidification (*n* = 5/group). (**D**) Mean *I*_NBC_. (**E**) Representative *I*_Cl_ and *I*_NBC_ mediated by the chimeric transporter. *I*_Cl_ is absent while *I*_NBC_ is produced. One of nine recordings is shown. (**F**) *I–V* relationships of the TM5 chimeric transporter and D555E. Currents were obtained in Cl^−^-free ND96 (*open markers*) and 1 min after switching to a solution containing 71 mM Cl^−^ (*closed markers*). Data were averaged from 5 oocytes per group. (**G**) Mean Cl^−^ conductance, *G*_Cl_. *G*_Cl_ was calculated from slopes in *I*_Cl_–V curve, which is the difference in *I*–*V* relationships between the presence and absence of Cl^−^ in (**F**). Slopes were measured near zero-current potentials; ** *p* < 0.01.

D555E is charge-conserved but has different geometry of the carboxyl group in the side chain due to an additional carbon backbone. This led us to postulate that Glu^555^ in D555E interacts with a nearby residue which results in a gain of function to select Cl^−^. To investigate this possibility, we replaced NBCe1/TM5 with NBCn1/TM5, which contains a Glu at the corresponding site of Asp^555^, and measured *I*_Cl_ in the chimeric transporter. First, we determined the functionality of the chimeric transporter by simultaneous recording of pH_i_ and *I*_NBC_ in voltage clamp (Figure 3A,B). In oocytes expressing NBCe1, the pH_i_ initially decreased upon CO_2_/HCO_3_^−^ application, due to CO_2_ influx followed by H^+^ accumulation from hydration (Figure 3A). The pH_i_ was then recovered from an acidification (*arrow*) as HCO_3_^−^ is continuously transported into the oocyte by NBCe1 and associates with intracellular H^+^. Applying CO_2_/HCO_3_^−^ also elicited an outward *I*_NBC_ (*arrowhead*), consistent with an influx of a net negative charge, due to 1 Na^+^ and 2 HCO_3_^−^ (or 1 CO_3_^2−^). Figure 3B is a recording of pH_i_ and *I*_NBC_, produced by the TM5-replaced chimeric transporter, subjected to the same experimental protocol. The chimeric transporter had a slower pH_i_ recovery rate (dpH/dt) and smaller *I*_NBC_ in CO_2_/HCO_3_^−^ solution than NBCe1. Consistent with this observation, mean dpH/dt and *I*_NBC_ from 5 oocytes per group were significantly decreased in the chimeric transporter (*p* < 0.01 for each; Figure 3C,D). Despite such decreases, the chimeric transporter is functional, as it recovers pH_i_ from an acidification and produces *I*_NBC_. Next, we measured the *I*_Cl_ and *I*_NBC_ produced by the chimeric transporter. Interestingly, the chimeric transporter did not produce measurable *I*_Cl_, while retaining *I*_NBC_ (Figure 3E; one of 9 oocytes expressing the chimeric transporter is shown). Consistent with this result, *I*–*V* relationships exhibited negligible change in curves before and after Cl^−^ application (*p* > 0.05, *n* = 5; Figure 3F). Figure 3G is the comparison of Cl^−^ conductance (*G*_Cl_), calculated from the slope of the *I*_Cl_–*V* relationship (i.e., difference in *I–V* curve before and after Cl^−^ application). *G*_Cl_ of the chimeric transporter was negligible.

### 2.4. I_Cl_ Induced by Lys^558^ Replacement in the TM5 Chimeric Transporter

**Figure 4:**
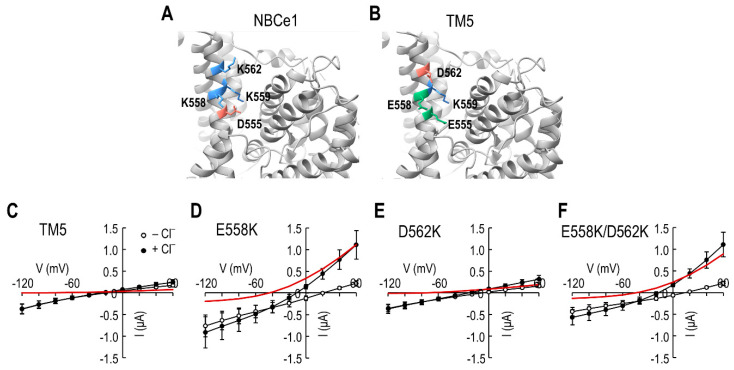
Lys^558^ replacement in the TM5 chimeric transporter induces *I*_Cl_. (**A**) Anion binding site S2 in NBCe1. Site S2 is constituted with Asp^555^, Lys^558^, Lys^559^, and Lys^562^. (**B**) Amino acid residues at the corresponding positions in the TM5 chimeric transporter. Glu^555^, Glu^558^, Lys^559^, and Asp^562^ are present at the site equivalent to S2. (**C**–**F**) I–V relationships of point mutant transporters for *I*_Cl_. E558K, D562K, and E558K/D562K are point mutants constructed on the chimeric transporter. The difference between the two mean is *I*_Cl_ (red line). Data were averaged from 4–5 oocytes per group.

The result of negligible *I*_Cl_ in the chimeric transporter indicates that Glu^555^ is not the sole residue for *I*_Cl_ and additional residues are involved. Those residues should be in TM5 because other TMs were unchanged in the chimeric transporter. Asp^555^ is a residue in the anion binding site S2 that includes Lys^558^, Lys^559^, and Lys^562^ (Figure 4A). The chimeric transporter contains Glu^555^, Glu^558^, Lys^559^, and Asp^562^ at the corresponding sites (Figure 4B), suggesting that residues at position 558 and 562 would be responsible for *I*_Cl_. To test this possibility, we changed Glu^558^ and Asp^562^, individually or together, in the chimeric transporter with a Lys and tested their ability to produce *I*_Cl_ (*n* = 4–5/group). Figure 4C shows the *I–V* relationships for the chimeric transporter without mutation. As expected, no significant difference was observed in the *I–V* curves before and after Cl^−^ application (red line in the figure). In contrast, replacing Glu^558^ with a Lys (E558K) increased the slope in the outward direction upon Cl^−^ application (Figure 4D). Replacing Asp^562^ with a Lys (D562K) had no effect (Figure 4E) and displayed similar *I–V* curves as the chimeric transporter. Consistent with these results, replacing both Glu^558^ and Asp^562^ with Lys (E558K/D562K) increased the slope in the outward direction upon Cl^−^ application (Figure 4F). Thus, Lys^558^ is responsible for producing *I*_Cl_.

**Figure 5:**
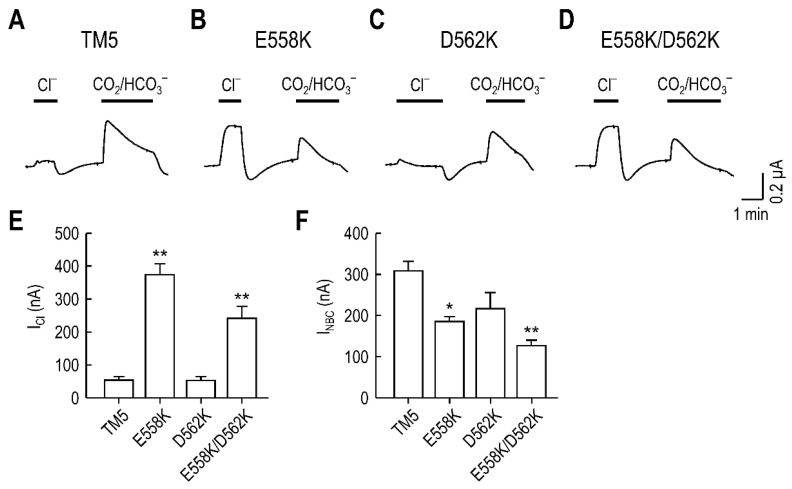
*I*_Cl_ induced by Lys^558^ replacement occurs without improving *I*_NBC_. (**A**–**D**) Representative I_Cl_ and I_NBC_ produced by the TM5 chimeric transporter (**A**), E558K (**B**), D562K (**C**), and E558K/D562K (**D**). *I*_Cl_ and *I*_NBC_ were recorded using the protocol described in Figure 1. (**E**) Mean *I*_Cl_ produced by the mutants. The level of significance was determined using one-way ANOVA, with Sidak post-test (*n* = 5–6/group). (**F**) Mean *I*_NBC_ produced by the mutants. Peak currents after CO_2_/HCO_3_^−^ application were measured. * *p* < 0.05 and ** *p* < 0.01 compared to TM5.

Next, we compared *I*_Cl_ and *I*_NBC_ produced by the mutant transporters. The chimeric transporter without mutation had negligible *I*_Cl_ but produced measurable *I*_NBC_ (Figure 5A). A transitional undershoot after Cl^−^ washout is probably due to endogenous Cl^−^ efflux which often occurs in some preparations of oocytes. E558K and E558K/D562K produced *I*_Cl_ (Figure 5B,D), whereas D562K did not (Figure 5C). Both E558K and E558K/D562K showed higher *I*_Cl_ amplitudes than *I*_NBC_ amplitudes, the reason of which is unclear. Comparison of mean *I*_Cl_ from 5–6 oocytes per group is summarized in Figure 5E. A significant amount of *I*_Cl_ was produced when a Lys was present at position 558 (*p* < 0.01, one-way ANOVA). We also compared mean *I*_NBC_ between groups to evaluate the effect of Lys mutations on Na/HCO_3_ cotransport and found a decrease in *I*_NBC_ by the mutations (*p* < 0.01, one-way ANOVA; Figure 5F). Thus, positively charged Lys residues in site S2 appear to have negative effects on the transporter activity.

### 2.5. Salt Bridge between Glu^555^ and Lys^558^

**Figure 6:**
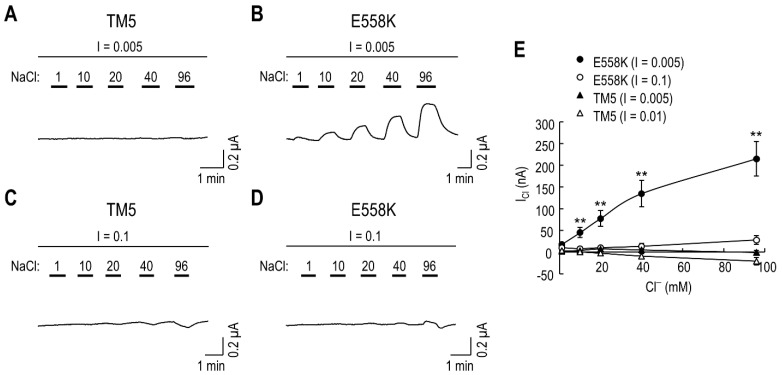
*I*_Cl_ is affected by solution ionic strengths. (**A**,**B**) Representative *I*_Cl_ evoked in solutions with the ionic strength (I) of 0.005 mol/L. An oocyte expressing the TM5 chimera (**A**) or E558K (**B**) was superfused with the low I solution in voltage clamp and exposed to a series of test solutions containing 1–96 mM of NaCl. Each test solution was bracketed with the low I solution to maintain steady-state baseline between test solutions. (**C**,**D**) Representative *I*_Cl_ evoked in solutions with I = 0.1 mol/L. Test solutions were applied to an oocyte expressing the TM5 chimera (**C**) or E558K (**D**) while the I was maintained using Na/gluconate. Each test solution was bracketed with Na/gluconate. (**E**) Comparison of *I*_Cl_ produced by the mutants. *I*_Cl_ was plotted as a function of Cl^−^ concentration *(n* = 4–6/group); ** *p* < 0.01 compared to TM5 (I = 0.005).

The identification of Lys^558^ for *I*_Cl_ leads to the possibility of a charge interaction between Glu^555^ and Lys^558^. To test whether a salt bridge stability is involved, we compared *I*_Cl_ produced by E558K in solutions containing either low or high ionic strength. The solution osmolarity was maintained using mannitol. The chimeric transporter displayed negligible response to 1–96 mM Cl^−^ in superfusing solutions, with the ionic strength of 0.005 and 0.1 mol/L (Figure 6A,C), consistent with its lack of *I*_Cl_. In contrast, E558K produced *I*_Cl_ with progressively larger amplitudes at higher NaCl concentrations when measured in solutions with the ionic strength of 0.005 mol/L (Figure 6B) but had nearly negligible *I*_Cl_, when measured in solutions with the ionic strength of 0.1 mol/L (Figure 6D). The result is consistent with the fact that a favorable salt bridge is diminished by a high ionic strength [21]. The decreasing effect by a high ionic strength was evident from the graph of *I*_Cl_ plotted as a function of Cl^−^ concentration (Figure 6E). The result shows effective inhibition of E558K-mediated *I*_Cl_ by a high ionic strength (*F*_12,80_ = 7.47, *p* < 0.01 for transporter x Cl^−^ concentration interaction, two-way ANOVA; *n* = 4–6/group).

### 2.6. Glu^555^–Lys^558^ Charge Interaction

**Figure 7:**
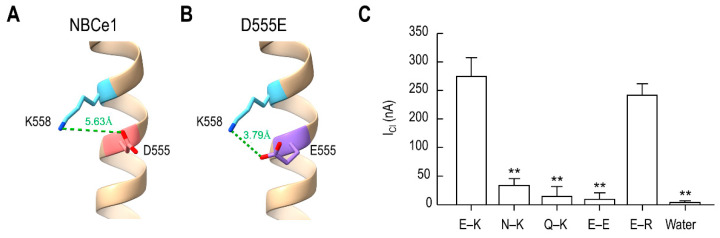
A charge interaction between Glu^555^ and Lys^558^ induces *I*_Cl_. (**A**,**B**) Bond length between the carboxyl group in the side chain of Asp^555^ (**A**) or Glu^555^ (**B**) and the amino group of Lys^558^. The length in angstrom was determined using the molecular visualization program ChimeraX with the rotamer probability of higher than 0.05. A hydrogen bond was identified when the bond length was <4 Å. (**C**) Comparison of *I*_Cl_ produced by mutations of Glu^555^ and Lys^558^. E–K is the Glu^555^–Lys^558^ pair (*n* = 7). N–K and Q–K are the replacement of Glu^555^ with an asparagine and a glutamine, respectively (*n* = 5/group). E–E and E–R are the replacement of Lys^558^ with an aspartic acid and an arginine, respectively (*n* = 4–5/group). Controls were water-injected oocytes (*n* = 4). ** *p* < 0.01 compared to E–K.

To further determine the above salt bridge interaction, we analyzed the bond length between the carboxyl group in the side chain of Glu^555^ and the amino group of Lys^558^ using the structure editing function with Dunbrack rotamer library [22] built in ChimeraX. In NBCe1, the distance between the carboxyl group of Asp^555^ and the amino group of Lys558 was 5.63 Å (Figure 7A), higher than the maximum 4.0 Å required for a hydrogen bond [23]. However, in D555E, the bond length between Glu^555^ and Lys^558^ was 3.79 Å (Figure 7B). The lengths from Lys^559^ and Lys^562^ were higher than 4.0 Å (data not shown). Thus, the bond length was consistent with a weak electrostatic interaction between Glu^555^ and Lys^558^, but neither Lys^559^ nor Lys^562^. The importance of the Glu^555^/Lys^558^ interaction for *I*_Cl_ was further examined by replacing Glu^555^ and Lys^558^ residues with other amino acids and comparing their ability to produce *I*_Cl_ (Figure 7C). Replacing Glu^555^ with a neutral Asn or Gln (N–K and Q–K pairs in the figure) or Lys^558^ with an Asp (E–E) near completely abolished *I*_Cl_. In contrast, replacing Lys^558^ with a positively charged Arg (E–R) retained measurable *I*_Cl_. One-way ANOVA, with Sidak post-test, revealed a significant change in *I*_Cl_ for N–K, Q–K and E–E pairs compared to E–K and E–R pairs (*F*_5,24_ = 25.49, *p* < 0.01; *n* = 4–7/group). Water-injected control showed no current.

## 3. Discussion

In this study, we examined the effects of Asp/Glu^555^ and other charged residues in the entrance anion binding site S2 on Cl^−^ selectivity and made the following observations. (i) Replacing Asp^555^ in NBCe1 with a charge-conserved Glu induces a permissiveness to Cl^−^ that is normally not a substrate. This replacement does not alter HCO_3_^−^ selectivity as *I*_NBC_ is favorably produced when both HCO_3_^−^ and Cl^−^ are present. (ii) Under the Na^+^-free condition, D555E produces *I*_Cl_ even if HCO_3_^−^ is available in the bath. The reason is that the anion binding site is not occupied with HCO_3_^−^ in this condition; as a result, Cl^−^ is accessible to the site. Thus, Na^+^ is required for HCO_3_^−^ to access its binding site. (iii) The *I*_Cl_ induced by D555E is due to a charge interaction between Glu^555^ and Lys^558^. Other Lys residues in site S2 have negligible effects on Cl^−^ transport. Glu^555^ and Lys^558^ are not simultaneously present in any member of the SLC4A bicarbonate transporters, indicating that the high HCO_3_^−^ selectivity in these transporters is maintained by avoiding a charge interaction between the two residues. This molecular feature is interesting as it is generally understood that electrostatic interactions contribute to protein structure and create a suitable environment for protein function such as enzyme catalysis, protein-ligand binding, thermal stability, and macromolecular assemblies [21,24,25]. In this sense, our study provides novel evidence that the anion selection in the bicarbonate transporters is established by avoiding a specific interaction between residues in the anion binding site, rather than maintaining such interaction.

The amino acid residues in the chimeric transporter we examined correspond to Asp^555^, Lys^558^, and Lys^562^ in NBCe1, all of which constitute site S2 located near the entrance of the ion accessibility pathway. Site S2 also contains Lys^559^, a DIDS-interacting residue [12], but we did not examine this residue for Cl^−^ selectivity because it is conserved in all SLC4A transporters. The SLC4A transporters contain either Asp^555^ or Glu^555^ but maintain a high selectivity to HCO_3_^−^; thus, a residue capable of interacting with these residues should not be conserved. The CryoEM of NBCe1 [11] shows that Asp^555^ and Lys^558^ are located to the protein center, while Lys^559^ and Lys^562^ are positioned further away from the center. The bond length between Asp^555^ and Lys^558^ is higher than the maximum length required for a salt bridge to take place, but Glu^555^ substitution has decreased the length (Figure 7A,B). We have previously demonstrated that D555E produces a large conductance in response to NO_3_^−^, which is structurally in a trigonal planar arrangement. The effective radius of NO_3_^−^ is bigger than the molecular radius of Cl^−^ (1.89 Å for NO_3_^−^ vs. 1.81 Å for Cl^−^), but D555E produces a larger NO_3_^−^ current than *I*_Cl_. It is, thus, likely that site S2 is molded to sterically distinguish HCO_3_^−^ or CO_3_^2−^ from other polyatomic anions in a trigonal planar arrangement. The charge interaction between Glu^555^ and Lys^558^ in D555E modifies this steric arrangement in a way that other structurally similar ionic compounds, including NO_3_^−^, are allowed. The modified steric arrangement also allows Cl^−^ to access the site but, given its monatomic molecule and competition with HCO_3_^−^ or CO_3_^2−^, we think that a Cl^−^ leak occurs at one of the three coordinating residues for peripheral oxygen atoms of HCO_3_^−^ or CO_3_^2−^. This interpretation is consistent with the MD simulations that Lys^558^ and Lys^559^ are the closest coordinating residues of CO_3_^2−^, determined from ion density maps and contact frequency analysis.

The results from our study provide new insights into the mechanism underlying ion transport in NBCe1, in addition to anion selectivity. The Glu^555^–Lys^558^ pair produces *I*_Cl_ in the absence of CO_2_/HCO_3_^−^ and *I*_NBC_ in the presence of CO_2_/HCO_3_^−^; that is, the presence or absence of *I*_Cl_ reflects whether the anion binding site is occupied with HCO_3_^−^ or CO_3_^2−^. *I*_Cl_ is produced in Na^+^-free CO_2_/HCO_3_^−^; thus, the binding site is not occupied in the absence of Na^+^, indicative of Na^+^ precondition prior to anion binding. Based on this interpretation, a model of the ion binding process can be made. In NBCe1, ion transport begins with a recruitment of Na^+^ to its binding site. The Na^+^ binding then allows HCO_3_^−^ or CO_3_^2−^ to access its anion binding site and as a result both ions are bound to the transporter. The steric arrangement of Asp^555^, Lys^558^, and Lys^559^ in site S2 is critical for distinguishing HCO_3_^−^ or CO_3_^2−^ from other anions. The same ion recruitment process also takes place in a mutant transporter containing the Glu^555^–Lys^558^ pair, such as D555E. However, the charge interaction between the two residues modifies the steric arrangement of residues in S2, such that other anions, such as Cl^−^, are permissive; as a result, Cl^−^ is accessible to the anion binding site. Our model proposes that Na^+^ binding is a necessary first step prior to anion binding and, thus, should be independent of external HCO_3_^−^ or CO_3_^2−^ levels. In this sense, it is interesting to note that the apparent affinity of NBCe1 for Na^+^ is independent of external HCO_3_^−^ concentrations [26]. We think that the negatively charged Asp/Glu^555^ facilitates Na^+^ recruitment from the extracellular fluid surrounding the transporter. One might argue that Na^+^ should overcome an electrostatic repulsion from the positively charged Lys residues before reaching its binding site. Decreased *I*_NBC_ by E5558K and E558K/D562K (Figure 5F) could be accounted for by the electrostatic repulsions from Lys residues. On the other hand, Yamazaki et al. [27] have reported that K558R, a single nucleotide polymorphism in human NBCe1, has a significantly reduced transport activity but no change in apparent Na^+^ affinity. Either way, it is premature to conclude that Lys residues influence Na^+^ recruitment to the binding site.

The MD simulation model proposes that substrate ions transiently bind to site S2 and then move to site S1, which ultimately leads to a protein conformational change for ion translocation. The TM5-replaced chimeric transporter in this study contains NBCn1-S2 but still produces *I*_NBC_, indicative of electrogenic cotransport. Thus, *I*_NBC_ can be induced, regardless of whether site S2 is molded for HCO_3_^−^ transport in NBCn1 or HCO_3_^−^ or CO_3_^2−^ transport in NBCe1. Then, a question arises whether the charges in site S2 are critical for HCO_3_^−^ or CO_3_^2−^ recruitment. The chimeric transporter contains NBCe1-S1, indicating that the production of *I*_NBC_ is determined by the anion that occupies site S1. We think that, whereas site S2 allows a transient binding of HCO_3_^−^ or CO_3_^2−^, S1 determines which of the two anions is translocated via the transporter. Our interpretation further suggests that NBCn1-S2 can recruit CO_3_^2−^, in addition to HCO_3_^−^, although HCO_3_^−^ is more favorably recruited. Nevertheless, it is difficult to envision how CO_3_^2−^ is selected by both NBCn1-S2, which contains negatively charged residues, and NBCe1-S2, which contains positively charged residues. Additional studies are demanded to elucidate the role of site S2 in anion recruitment.

Does *I*_Cl_ induced by the Glu^555^–Lys^558^ pair represent a channel activity or transporter activity? If *I*_Cl_ is a channel activity, we should then observe a current in response to HCO_3_^−^ (*I*_HCO3_), comparable to *I*_Cl_ in response to Cl^−^. However, we did not observe *I*_HCO3_ under the Na^+^-free condition. The lack of *I*_HCO3_ under the Na^+^-free condition reflects that HCO_3_^−^ movement via D555E is solely mediated by electrogenic Na/HCO_3_ transport that generates *I*_NBC_. The important finding is that *I*_Cl_ is significantly inhibited by electrogenic Na/HCO_3_ transport (Figure 1B,C), indicating that *I*_Cl_ competes with *I*_NBC_. Thus, *I*_Cl_ is associated with a transporter activity. As described above, we envision that D555E modified the HCO_3_^−^ binding site to produce an anion leak. On the other hand, *I*_Cl_ can be produced without Na^+^, implicating a separate channel activity. This leads us to a conclusion that *I*_Cl_ is associated with both transporter activity and channel activity, and they overlap. It is difficult to envision how the two activities overlap, and additional studies are required to address the exact nature of *I*_Cl_.

Lastly, our study leads us to a discussion about a pathological implication of Cl^−^ leak mediated by mutations in NBCe1. Cl^−^ and HCO_3_^−^ movements tightly coordinated in many cells, and specific transporters and channels are involved in regulating such coordination [28,29,30,31]. Obviously, Cl^−^ leak is undesirable in cells and tissues where NBCe1 is highly expressed and regulates HCO_3_^−^ transport for cellular and physiological function. Myers et al. [32,33] have reported that Q913R, a mutation identified from a patient with proximal renal tubular acidosis, causes intracellular retention of NBCe1 and a gain of function activity in Cl^−^ leak. It is expected that this mutation causes a depolarization in the basolateral membrane of renal proximal tubules; as a result, the driving force for HCO_3_^−^ reabsorption is decreased. A Cl^−^ leak via the mutation is also expected to alter the coupling of Cl^−^ and HCO_3_^−^ movement observed in secretory epithelia, such as pancreas and salivary glands [34]. Another mutation of interest is K558R that has a reduced transport activity [27]. Our analysis of the bond length between Asp^555^ and Arg^558^ in K558R is less than 4 Å (3.82 Å with the probability of 0.1 and 3.5 Å with the probability of 0.05), implicating a salt bridge between the two residues. It will be interesting to examine whether this mutation can cause Cl^−^ leak. Additionally, depending upon NBCe1 variants, intracellular Cl^−^ can regulate the transporter activity [35]. Thus, the lack of Cl^−^ leak in NBCe1 is beneficial for cellular HCO_3_^−^ homeostasis and epithelial electrolyte secretion.

In summary, by analyzing the TM5 chimeric transporter and relevant point mutants, we identified a charge interaction in site S2 as a key factor for anion selectivity and provided new insights into CO_3_^2−^ or HCO_3_^−^ recruitment to the binding site and ion binding sequence. Future studies will be of the molecular mechanism underlying ion selectivity and translocation in other Na/HCO_3_ transporters.

## 4. Materials and Methods

### 4.1. TM5-Replaced Chimeric Transporter and Point Mutants

D555E made on human NBCe1-A (Genbank accession number: NM_003759; hereafter, NBCe1) was described previously [17]. The TM5-replaced chimeric transporter was constructed by creating restriction enzyme sites at the TM5 boundaries in NBCe1 and NBCn1. Point mutant transporters were constructed using the QuickChange Site-directed Mutagenesis kit (Agilent Technologies, Santa Clara, CA, USA). Primers were designed to replace nucleotides encoding candidate amino acids (Supplementary Table S1). PCR was carried out 95 °C for 1 min, 55 °C for 30 s, and 68 °C for 10 min for 16 cycles, and an additional 2 min per nucleotide substitution were included for extension at 68 °C. All constructs were sequenced.

### 4.2. Protein Expression in Xenopus Oocytes

*Xenopus laevis* oocytes, at stages V-VI, were purchased from Ecocyte Bioscience (Austin, Texas, USA). For cRNA synthesis, plasmids containing NBCe1 or mutant transporter DNAs were linearized and transcribed using the mMessage/mMachine transcription kit (Life Technologies, Grand Island, NY, USA). Transcribed RNAs (15–25 ng in 46 nL) were injected per oocyte. Equal amounts of RNAs were used when multiple samples were compared. Controls were water injection. Oocytes were maintained for 3–4 days at 18 °C before use.

### 4.3. Two-Electrode Voltage Clamp

An oocyte was placed in the recording chamber containing ND96 solution (in mM; 96 NaCl, 2 KCl, 1.8 CaCl_2_, 1 MgCl_2_, and 10 HEPES, pH 7.4) and impaled with two borosilicate glass electrodes filled with 3 M KCl. The tip resistance was 0.5–2 MΩ. After stabilization of the resting membrane potential, the oocyte was clamped at –60 or 0 mV using the voltage-clamp amplifier OC-725C (Warner Instrument, Hamden, CT, USA). For recording *I*_Cl_, an oocyte was superfused with Cl^−^-free solution, which replaced all NaCl in ND96 solution with Na/gluconate, and then with 71 mM Cl^−^ solution, which replaced 25 mM NaCl with the same amounts of Na/gluconate. A small amount of Cl^−^ (<3 mM) was included to minimize junction potential. For recording *I*_NBC_, 25 mM NaCl was replaced with the same amounts of NaHCO_3_ equilibrated with 5% CO_2_. Recording *I*_Cl_ in CO_2_/HCO_3_^−^ solution was achieved after *I*_NBC_ reached steady-state. Na^+^-free solutions were made by substituting Na^+^ with N-methyl-D-glucamine NMDG. Current-voltage (*I–V*) relationships were determined by a staircase voltage command between −120 to +60 mV, with 20 mV increments for 100 ms duration. The voltage command was applied immediately after a current reached steady state. Voltage signals were sampled by Digidata 1322A (Molecular Devices; San Jose, CA, USA) and data were acquired using pClamp 10 (Molecular Devices). Signals were filtered using an 8-pole low pass Bessel filter, with a cutoff frequency of 0.1–1 Hz. Recordings were made at room temperature.

### 4.4. Measurement of Intracellular pH (pH_i_)

Oocyte pH_i_ was measured using a proton-selective glass electrode, as described before [36]. Briefly, a pH electrode was made with a borosilicate glass capillary that was silanized, filled with the proton ionophore 1 cocktail B (cat no: 95293, MilliporeSigma, St. louis, MOP, USA), and back-filled with pH 7.0 phosphate buffer. The pH electrode was connected to high impedance electrometer FD223 (World Precision Instruments; Sarasota, FL, USA) and electrode signals were amplified using a custom-made subtraction amplifier. Current and voltage electrodes were filled with 3 M KCl (resistance of 0.5–2 MΩ) and connected to an OC-725C amplifier. Signals from pH, current, and voltage electrodes were collected using Digidata 1322A. The voltage electrode signal was subtracted from the pH electrode signal using pClamp 10. The voltage/pH slope was calibrated by placing electrodes in the chamber filled with pH 6.0 and 8.0 standards. Slopes were typically at the range of 53 ± 3 mV/pH. An oocyte, in the recording chamber containing ND96 solution, was impaled with pH, voltage, and current electrodes and clamped at 0 mV. Once pH and base line current were stabilized, solutions were switched to 5% CO_2_, 25 mM HCO_3_^−^ (pH 7.4). The rate of pH change (dpH/dt) was determined by drawing a line during the first 4 min of recovery from CO_2_-induced acidification.

### 4.5. Salt Bridge Experiment

For assessment of salt bridges, an oocyte expressing the mutant transporters was clamped at 0 mV and superfused with 96 mM Na/gluconate (plus 5 mM mannitol) or 197 mM mannitol, plus a small amount of chloride (<3 mM), until base line currents became stable. Then, a series of test solutions containing 1, 10, 20, 40, and 96 mM of NaCl were applied. NaCl in each test solution replaced the equivalent amount of mannitol or Na/gluconate. Each test solution was bracketed with NaCl-free solution to maintain steady-state baseline between test solutions. The ionic strength (I) was determined using the equation:

$$I=\frac{1}{2}\sum_{i=1}^{n}CiZi^{2},$$

where C*i* is the molar concentration of ion *i* (mol/L), and Z*i* is the charge number of that ion.

### 4.6. Analysis of Charge Interaction in Site S2

Analysis of the binding site S2 was performed with CryoEM structure of the human NBCe1 (accession code: 6CAA) from the RCSB Protein Data Bank using the molecular visualization program UCSF ChimeraX 1.1 [37]. A hydrogen bond between the side chain carboxy group of Asp^555^ and amino group of nearby Lys residues was identified when the distance between them was <4 Å. For D555E or the TM5-replaced chimeric transporter, amino acid changes were analyzed using the structure editing function with Dunbrack rotamer library [22] built in ChimeraX. A hydrogen bond was identified from the rotamer probability of higher than 0.05.

### 4.7. Statistical Analysis

Data were reported as mean ± standard error. The level of significance was determined using (i) unpaired, two-tailed Student t-test for comparison between NBCe1 and D555E or chimeric protein; (ii) paired, one-tailed test for comparison of single transporters in two different solutions; (iii) one-way ANOVA for comparison of *I*_Cl_ or *I*_NBC_ among multiple mutants; and (iv) two-way ANOVA for comparison between *I*_Cl_ vs. *I*_NBC_ among multiple mutants. The *p* value of less than 0.05 was considered significant. Data were analyzed using Prism 7 (GraphPad; La Jolla, CA, USA) and Microsoft Office Excel add-in Analysis ToolPak (Redmond, WA, USA).

## Data Availability

The data presented in this study are available upon request to I.C.

## Supplementary Materials

The following is available online at https://www.mdpi.com/article/10.3390/ijms23010532/s1.
