# Supplementary material for: Lack of Charge Interaction in the Ion Binding Site Determines Anion Selectivity in the Sodium Bicarbonate Cotransporter NBCe1
Source: Int J Mol Sci. 2022 Jan 4;23(1):532. doi: 10.3390/ijms23010532 (PMC8745181; doi:10.3390/ijms23010532)
Supplement: Supplementary file 1 [file ijms-23-00532-s001.zip › ijms-1490245-supplementary.pdf]

Table S1. Primer sequence used to construct mutant transporters

Primer	Sequence (5′ to 3′)
D555E	Forward: ATCTTTATCTATGAAGCTTTCAAGAAG<br>Reverse: CTTCTTGAAAGCTTCATAGATAAAGAT
TM5/NBCe1	Forward: GATCAAGCTTGCATGCTACTACCCCATC<br>Reverse: GATGGGGTAGTAGCATGCAAGCTTGATC
TM5/NBCn1	Forward: GCTCTTTGATTTAGCATGCACATATGC<br>Reverse: GCATATGTGCATGCTAAATCAAAGAGC
E558K (E–K)	Forward: CTACGAGGCTTTGAAGAAGCTCTTTGATTTAGCAGATTACTACC<br>Reverse: GGTAGTAATCTGCTAAATCAAAGAGCTTCTTCAAAGCCTCGTAG
D562K	Forward: CTACGAGGCTTTGGAGAAGCTCTTTAAGTTAGCAGATTACTACC<br>Reverse: GGTAGTAATCTGCTAACTTAAAGAGCTTCTCCAAAGCCTCGTAG
E558K/D562K	Forward: CTACGAGGCTTTGAAGAAGCTCTTTAAGTTAGCAGATTACTACC<br>Reverse: GGTAGTAATCTGCTAACTTAAAGAGCTTCTTCAAAGCCTCGTAG
N–K	Forward: ATCTTTATCTATAATGCTTTCAAGAAG<br>Reverse: CTTCTTGAAAGCATTATAGATAAAGAT
Q–K	Forward: CATCATATTCATCTACCAGGCTTTGAAGAAGCTC<br>Reverse: GAGCTTCTTCAAAGCCTGGTAGATGAATATGATG
E–R	Forward: CTACGAGGCTTTGAGGAAGCTCTTTGATTTAG<br>Reverse: CTAAATCAAAGAGCTTCCTCAAAGCCTCGTAG
